# Oncolytic virotherapy in veterinary medicine: current status and future prospects for canine patients

**DOI:** 10.1186/1479-5876-10-3

**Published:** 2012-01-04

**Authors:** Sandeep S Patil, Ivaylo Gentschev, Ingo Nolte, Gregory Ogilvie, Aladar A Szalay

**Affiliations:** 1 Department of Biochemistry, University of Wuerzburg, D-97074 Wuerzburg, Germany; 2 Genelux Corporation, San Diego Science Center, San Diego, California, USA; 3 Small Animal Clinic, University of Veterinary Medicine, D-30173 Hannover, Germany; 4 Angel Care Cancer Center, California Veterinary Specialists, Carlsbad, CA, 92008, USA; 5 Department of Radiation Oncology, Moores Cancer Center, University of California, San Diego, 3855 Health Sciences Drive # 0843, La Jolla, CA 92093, USA; 6 Rudolf Virchow Center for Experimental Biomedicine, University of Wuerzburg, D-97078 Wuerzburg, Germany; 7 Institute for Molecular Infection Biology, University of Wuerzburg, D-97078 Wuerzburg, Germany

**Keywords:** cancer, canine cancer therapy, oncolytic virus, oncolysis, target molecule, combination therapy

## Abstract

Oncolytic viruses refer to those that are able to eliminate malignancies by direct targeting and lysis of cancer cells, leaving non-cancerous tissues unharmed. Several oncolytic viruses including adenovirus strains, canine distemper virus and vaccinia virus strains have been used for canine cancer therapy in preclinical studies. However, in contrast to human studies, clinical trials with oncolytic viruses for canine cancer patients have not been reported. An 'ideal' virus has yet to be identified. This review is focused on the prospective use of oncolytic viruses in the treatment of canine tumors - a knowledge that will undoubtedly contribute to the development of oncolytic viral agents for canine cancer therapy in the future.

## 1. Introduction

Cancer is the most common cause of natural death in dogs and endemic in both developed and developing countries [1,2]. Incidence of cancer ranges from 1 to 2% in the canine population and currently accounts for about half of the deaths in dogs older than 10 years [1,3]. The major treatment options for canine cancers include surgery, radiation therapy, chemotherapy, hyperthermia and photodynamic therapy. As the standard therapy is usually palliative in canine cancer, there is an excellent opportunity to evaluate alternative approaches.

A promising therapeutic approach is the oncolytic virotherapy. Oncolytic viruses selectively infect, replicate and kill cancer cells, while leaving healthy cells intact. Evidence that viruses may be useful in the eradication of cancer has existed since the early twentieth century [4,5]. These early discoveries have led to the testing of several viruses against cancers in both pre-clinical and clinical settings during the 1950s and 1960s [6]. Alice Moore was the first scientist to test oncolytic virotherapy in an animal model. Working with Russian Far East encephalitis virus, complete regression was achieved in some cases of mouse sarcoma 180 - the first animal model to demonstrate full regression through viral oncolysis [7]. However, during the last 15 years numerous reports have confirmed that intratumorally or systemically delivered viruses such as Newcastle disease virus (NDV) [8], reovirus [9], lentivirus [10] herpes simplex virus (HSV) [11], enterovirus [12], Sindbis virus [13], Semliki Forest virus [14] Seneca Valley virus [15], adenovirus [16], vaccinia virus [17], myxoma [18] and raccoonpox virus [19] could display an antitumor activity in different animal models (for more detailed list of oncolytic viruses readers are directed to [20-22]). Several oncolytic virus (OV) platforms (herpes simplex virus, vaccinia virus, Seneca valley virus and reovirus) are currently in or entering Phase III human clinical trials. In addition, in China the oncolytic adenovirus H101 has been approved in the treatment of human patients with head and neck cancer since 2005 [23].

However, currently the use of oncolytic virotherapy in veterinary medicine is far from reality and promising laboratory results have to be translated into improved clinical outcomes. This review describes the most common classes of oncolytic viruses for canine cancer therapy, and focuses on ways in which these viruses may be manipulated in order to target, enhance and exploit their cytolytic properties for treatment of canine cancer.

## 2. Oncolytic Virotherapy for Canine Cancer

Oncolytic viruses such as various human and canine adenoviruses, canine distemper virus (CDV) and vaccinia virus strains have been preclinically tested for canine cancer therapy.

Adenoviruses are medium-sized (90-100 nm), nonenveloped icosohedral viruses containing double-stranded DNA. They are well characterised for oncolytic therapy in humans and have the ability to infect a broad range of cells cross-species [24,25]. These viruses are restricted to cancer cells at the replication level and hence called conditionally replicating adenoviruses (CRAds). E1A and E1B gene regions of wild type adenovirus are deleted, making virus replication competent in those cells in which pRb pathway or p53 apoptosis pathway is defective [26]. Moreover, control mechanisms have been added to adenoviruses to ensure cancer cell specificity, and arming the virus with suicide genes has also been explored to improve therapeutic effects [27]. Adenoviruses are also being tested as therapeutic agents for canine cancers. Human adenovirus 5 has been shown to productively replicate in canine osteosarcoma and canine mammary carcinoma cells [28]. Furthermore, canine adenovirus 2 (CAV-2), transcriptionally targeted to canine osteosarcoma cells by inserting osteocalcin promoter, was tested as therapeutic agent for canine osteosarcoma. As this promoter is active only in osteosarcoma cells and not active in other canine non neoplastic cells, CAV-2 with osteocalcin promoter showed restricted replication in canine osteosarcoma cells [29]. This modified canine adenovirus killed canine osteosarcoma cells in cell culture and showed therapeutic benefits in xenograft model [30]. Moreover, administration of CAV-2 to normal dogs showed no major signs of virus-associated toxicity [29]. Arming an adenoviral vector has yielded promising results in canine malignant melanoma cases. Intratumoral injections of this adenoviral vector with CD40 ligand (AdCD40L) revealed inhibition of tumor progression and metastasis in two canine malignant melanoma cases. Also, injections of AdCD40L were safe and no signs of systemic toxicity were observed in canine patients throughout the treatment period [31]. Extension of such studies to clinical trials in the canine population will likely provide better understanding of the oncolytic properties, safety, and therapeutic efficiency of adenoviruses.

Canine distemper virus (CDV) is an enveloped virus with a single stranded RNA genome belonging to the family *paramyxoviridae *[32]. It is a morbillivirus and possesses many common features with human Measles virus (MV) including its clinical outcomes [33]. Clinical consequences of CDV and MV include profound immune suppression and lymphopenia. Interestingly, regression of Hodgkin's disease was observed in children after concurrent MV infection [34,35] which prompted consideration of the use of attenuated CDV for treatment of canine lymphoma. Moreover, CDV binds to a cellular receptor, Signalling Lymphocyte Activation Molecule (SLAM or CD150) [36,37]. Canine lymphoid cell lines and B and T lymphocytes established from dogs with lymphoma have been shown to express CD150 receptors. Attenuated CDV has been tested for oncolytic property in the lymphoma cells and was able to infect and induce apoptosis in these cells [33]. It may therefore be used to treat canine lymphoma patients.

In addition, several preclinical and clinical studies with vaccinia virus (VACV) helped to determine its oncolytic property. VACV is a member of the poxvirus family characterised by double stranded DNA genome [38,39]. It was the first widely used vaccine (in over 200 million people) resulting in eradication of smallpox worldwide, and therefore much is known about its safety profile [40]. Two oncolytic vaccinia virus strains, namely JX-594 (Jennerex Biotherapeutics, Inc. USA) and GLV-1h68 (Genelux Corporation, USA), have shown promising preclinical data and are now undergoing clinical trials in humans [17,41,42]. The JX594 construct is based on a thymidine kinase (*tk*) gene-deleted strain derived from the Wyeth strain and expresses GM-CSF and beta-galactosidase (LacZ) [43]. GLV-1h68 (denominated as GL-ONC1 when produced under GMP conditions) was developed on the base of the Lister strain and carries the gene sequences for a fusion protein of *Renilla *luciferase and Green Fluorescent Protein (Ruc-GFP), LacZ and beta-glucuronidase (GusA) [17]. In addition, GLV-1h68 has been proposed for the treatment of canine mammary adenoma and carcinoma [44,45]. The effect of this virus was studied *in vitro *in the canine mammary carcinoma cell line MTH52c and canine mammary adenoma cell line ZMTH3 and in xenograft models in nude mice. These studies revealed that GLV-1h68 could efficiently infect, replicate in and destroy canine mammary carcinoma MTH52c cells and canine mammary adenoma ZMTH3 cells in culture. Furthermore, in both cases, significant inhibition of tumor growth and damage of tumor tissues was observed after systemic administration of GLV-1h68 in tumor bearing nude mice [44,45]. Additionally, the opportunity to localize GLV-1h68 viruses via optical imaging might be utilized in metastasis detection [46]. Moreover, the oncolytic vaccinia virus strain LIVP 1.1.1 efficiently kills canine soft tissue sarcoma cells **(Gentschev I *et. al*., submitted)**. In this very recent study, a systemic administration of LIVP 1.1.1 virus, a new variant isolated from the wild-type LIVP strain **(Chen *et. al*, unpublished data)**, led to significant growth inhibition and regression of the tumors in a canine sarcoma xenograft mouse model **(Gentschev I *et. al *submitted)**. The LIVP 1.1.1 mediated therapy was further shown to be sufficient for long-term survival of sarcoma bearing mice and resulted in almost complete tumor regression.

Other poxviruses have also been explored as oncolytic agents for canine tumors. Pre-clinical studies with recombinant canary pox viruses (ALVAC) expressing interleukin 2 have shown the utility of these vectors in promoting anti-tumor activity [47]. Based upon these results, the effect of ALVAC was also analyzed clinically in canine cancer patients. Intratumoral administration of this recombinant poxvirus in dogs with melanoma revealed localized distribution of virus into tumor tissue [48]. Moreover, an ALVAC vaccine against canine distemper virus (RECOMBITEK rDistemper, Merial, USA), has been approved by the USDA and has been demonstrated to be safe and effective in preventing disease in practical field use. However, there are no data regarding the anti-tumor efficacy of ALVAC in canine patients. For a more detailed overview, the characteristics of several oncolytic viruses are listed in Table 1.

**Table 1:** Oncolytic viruses for canine cancer therapy

Virus	**Strains/ref**.	Advantages	Disadvantages
**Adenovirus**	CAV-1 [30] CAV-2 [29]	Infection of both dividing and non dividing cells. High viral titres in tumor tissue. Induction of immune response. Efficient gene transfer.	Pre-existing immunity in canine population. Small insert capacity.

**Canine** **Distemper** **Virus**	CDV [33]	Natural tumor tropism as cellular receptor for entry CD46 is expressed on tumor cells. Good safety records as included in vaccine schedule of canines.	Only lymphoma therapy data

**Vaccinia** **Virus strains**	GLV-1h68 [44,45]	Broad host range. Attenuated by three insertion cassettes. Large recombinant gene capacity. Efficient gene transfer and expression. No chance for integration of viral genome in to host.	Induction of virus-mediated immune response
	LIVP	Attenuated by natural mutation in thymidine kinase (tk) gene. Safety of virus is well known. Broad host range. Replication in host cytoplasm only, no chance for integration of viral genome in to host. Insertion of largest DNA fragment (about 25 kBps) for gene therapy.	Only tested for canine soft tissue sarcoma.

**Canary Pox virus**	ALVAC [47]	Producing an immune response without any adjuvant. Approved by the USDA as a vaccine against canine distemper virus (RECOMBITEK canine distemper)	Protection against canine distemper virus only

**Abbreviations: **CAV-1: Canine adenovirus - 1, CAV-2: Canine adenovirus - 2, CDV: Canine distemper virus, LIVP: Lister strain of Vaccinia virus, ALVAC: Recombinant canarypox virus, GLV-1h68: Recombinant Vaccinia virus by Genelux Corporation USA.

## 3. Current and Future Challenges of Oncolytic Viral therapy for Canine Cancers

Despite encouraging progress in the field of oncolytic viral therapy, several limitations remain in the development of an ideal OV including selective targeting of OVs to tumor tissue, relatively poor virus-spread throughout solid tumor tissue, inefficient viral replication in immune-competent hosts and disadvantageous ratio between anti-viral and anti-tumoral immunity. Several strategies to overcome these limitations are currently being investigated. The major strategies include the improvement of existing oncolytic vector systems or using combinations of oncolytic viruses with existing clinical methods or agents for synergistic and/or additive anti-tumor responses. Oncolytic virotherapy for canine cancers is still in its infancy; however, exciting developments in this pioneering approach are expected. This novel therapy has limitations which need to be addressed to improve efficacy, safety and clinical applicability. An overview of some of the problems and solutions is described here.

### 3.1 Selective targeting of oncolytic virus to tumor tissue

A major caveat for the widespread clinical use of virotherapy in canine cancer is to ensure that viruses do not harm normal cells. Certain viruses like NDV, reovirus, vesicular stomatitis virus (VSV), measles virus and others have natural tumor tropism [49-52]. However, better understanding of molecular events of virus-cell interactions in recent years has allowed for the design of genetically engineered viruses that target selected molecules or signalling pathways such as p16, p21 p53, IFN pathway, PTEN, EGFR, VEGFR, STAT3, HSP70, anti-apoptosis or hypoxia. Adenoviruses and vaccinia viruses are directed to human cancer cells by taking advantage of these defective pathways. Adenoviruses are designed to target human cancer cells mutated for tumor suppressor protein p53. The viral protein encoded by the E1 region of wild type adenovirus binds and inactivates p53, allowing replication of virus in normal cells [53]. Because tumor cells lacking functional p53 gene are unable to suppress replication of mutant adenoviruses, E1 gene-deleted adenoviruses have diminished ability to replicate in normal cell and preserved replication in neoplastic cells [26]. Canine p53 family proteins have biological activities similar to their human counterparts [54], with more than 85% gene sequence similarity. In addition, p53 mutations in canine tumors are located within the exons similar to those reported in human genes [55], and mutations in conserved domains of p53 appear to play a significant role in mammary carcinogenesis in both humans and dogs [56]. Like in human tumors, the p53 gene is mutated in several canine cancers, including osteosarcoma [57], mammary tumors [58] and gastric carcinoma [59]. All these points suggest that canine and human p53 protein has similar biological functions which can be helpful in designing better therapeutic modalities against canine cancer. Thus, oncolytic viruses, especially adenovirus, can specifically be targeted to canine cancer cells by taking advantage of the defective p53 pathway. In another example, VACV mutants with deletions in the thymidine kinase gene (*tk*) and/or vaccinia growth factor gene (VGF) are well advanced in pre-clinical and clinical studies for human cancers [60,61]. These mutants grow selectively in cancer cells with high levels of cellular thymidine kinase (TK), and constitutively activated EGFR/Ras pathway signalling complements the loss of the viral gene products [62]. Moreover, tumor cells release the TK enzyme to the circulation, probably due to disruption of dead or dying tumor cells [63]. Increased serum TK activity was observed in various canine malignancies like lymphoma [63,64], leukemia [65] and hemangiosarcoma [66], suggesting a possible target for directing Vaccinia virus to tumor tissue. In addition, higher expression of EGFR in mammary [67], glioma [68], hepatocellular carcinoma [69] and malignant epithelial nasal tumors [70] of canine origin closely parallels that of human tumors of the same type and histologic grade. High levels of EGFR and TK in various canine cancers could be basis of enhancing replication of Vaccinia virus in canine cancer cells.

To further enhance the tropism of vaccinia virus to human cancer cells, Kirn and colleagues deleted the B18R gene, which encodes a protein that neutralizes type I interferons (IFNs), producing a highly tumor-specific oncolytic vaccinia virus [71]. IFNs are a group of secreted cytokines, which exert pleiotropic effects on important cell functions, including cell proliferation and modulation of the immune system [72,73]. The IFN system also mediates the first line of cellular anti-viral response. Interestingly, about 70-75% of the cancer cells are defective in the IFN pathway [74]. Mutation in B18R gene allows the vaccinia virus to selectively infect (cancer) cells with defects in their IFN responses but not normal cells with intact IFN responses. In this context, canine cancer cells with defects of the IFN system may be optimal targets for OVs, which exploit such defects to support their own replication.

Numerous other strategies such as transductional targeting, which use conditionally replicating viruses e.g. canine adenovirus [30] and transcriptional targeting, which includes use of specific promoters [29], may also aid in selective targeting of oncolytic viruses to cancer cells.

### 3.2 Spread of oncolytic viruses throughout the tumor mass

Another challenge for effective oncolytic virotherapy is the relatively poor penetration of the virus throughout solid tumor masses. As observed in human cancers, the slow spread of virus in solid tumors can be limiting and determines the outcome of therapy [75]. The slow viral spread within solid tumors might relate to the relatively large size of OVs (e.g. around 200 nm of vaccinia virus and around 90 nm for adenovirus). Recently, Altomonte *et al*. showed that a single amino acid modification in the fusion protein of NDV greatly improved the fusogenicity of a recombinant virus, thereby resulting in enhanced tumor cell killing through the formation of large multi-nucleated syncytia and spread of the virus throughout the tumor mass [76].

The intratumoral spread and efficacy of OVs were also improved by protease or hyaluronidase mediated digestion of tumor extracellular matrix (ECM) [77-82]. Structural components of tumor ECM, such as collagens and proteoglycans, have been shown to hinder distribution of large therapeutic molecules and viruses [82]. Therefore, the degradation of extracellular matrix with OV expressing matrix proteases and collagenases may be a useful strategy to achieve anticancer effects in both humans and dogs.

### 3.3 Optimization of viral replication in immune-competent hosts

Immune responses against viruses presumably limit ongoing viral replication in immunocompetent dogs. In this context, a high level of pre-existing immunity to parental viruses in canine populations might limit the use of oncolytic viruses for cancer therapy. The role of virus-neutralizing antibodies following intravenous administration remains to be determined. Use of unrelated viruses from different hosts, such as vaccinia for dog cancers, may solve the problem of pre-existing immunity. However, carrier cell based therapy also provided promising results to escape pre-existing immunity [83]. In this regard, several types of cells, such as immune cells [84-86], stem cells [84,87,88] and tumor cells [89,90] were successfully utilized as carriers of oncolytic viruses to tumors. Specific delivery to tumors and escape of the pre-existing antiviral immunity increased the effective local viral dose in the tumor tissue and thus enhanced the oncolytic effects [90-92]. However, mechanisms for the specific homing of carrier cells to tumors are currently unknown.

In a recent study, canine osteosarcoma cells treated with replication selective canine adenovirus (OCCAV) were used as carrier vehicles for evading pre-existing neutralizing antibodies against adenovirus. Systemic antitumoral activity of OCCAV, even in presence of adenovirus neutralizing antibodies, suggests a promising approach to evade pre-existing immunity against the viral vector [93].

The efficiency of OV replication in tumor bearing immunocompetent dogs may be enhanced by various means such as combination of viro- with chemo- [94] or radiation therapy [95] or the conjunctive use of different oncolytic viruses [96].

### 3.4 Enhancing Anti-tumor Immunity and/or Anti-tumor Effects of OVs by Virus-integrated Genes

Several strategies have been developed to achieve better anti-tumoral immunity and/or anti-tumor effects after OV cancer treatment. One strategy involves the integration of genes encoding either proteins with immunomodulatory functions, such as cytokines or chemokines, or tumor associated antigens (TAA). However, the optimal balance between anti-viral and anti-tumor immune responses is crucial for the success of these cancer therapies in immunocompetent patients.

Another strategy to enhance the OV mediated anti-cancer activity is targeting the tumor microenvironment with replicating OVs. One promising target here is the tumor neoangiogenesis. Recently, Breitbach and colleges demonstrated that VSV directly infects and destroys tumor vasculature *in vivo*, leaving the normal vasculature intact [97]. In addition, armed oncolytic viruses can also prevent neoangiogenesis, leading to cancer cell necrosis. Vascular endothelial growth factor (VEGF) is a protein that plays a key role in tumor angiogenesis [98]. Vaccinia virus encoding anti-VEGFR-1 protein decreases neoangiogenesis at the tumor site and inhibits the tumor growth [99]. Furthermore, blocking VEGF has shown enhanced antitumor activity in a human xenograft model where vaccinia virus was armed with anti-VEGF antibody [100]. As seen for human tumors, significant expression of VEGF enhanced angiogenesis in canine mammary gland tumors [101]. Also, increased levels of VEGF-2 were observed in canine intracranial meningiomas [102], mammary adenocarcinoma [103], mastocytoma [104], mast cell tumors [105], and soft tissue sarcoma [106]. Expression of VEGF in a variety of canine cancers proves its role in canine tumor angiogenesis. Thus, oncolytic viruses armed with anti-VEGF agents will be a possible therapeutic approach for canine cancers.

Finally, the anticancer activity of OVs may also be improved by expression of prodrug-converting enzymes capable of producing a toxic product within tumor tissue. Recombinant oncolytic viruses have been used to express suicide genes that convert a pro-drug into toxic drug within the tumor. Nitro-reductase enzyme from *E. coli *causes reduction of inactive prodrug CB1954 to promote cell killing in feline cancer cells [107]. Similarly, 5-Fluorouracil is a pyrimidine analog widely used in canine cancer chemotherapy. Bacterial and/or yeast cytosine deaminase (CDase) is a well characterized enzyme-prodrug system that converts 5-Fluorocytosine to 5-Fluorouracil which further leads to cell cycle arrest and apoptosis leading to improved antitumor effects [108]. Therefore, OVs armed with nitroreductase or cytosine deaminase gene in combination with prodrugs may synergistically destroy canine cancer cells.

## 4. Combination of Oncolytic Viruses with Conventional Cancer Therapy in Dogs

One way to augment the efficacy of viral oncolysis is to combine genetically engineered replication competent viruses with standard anti-cancer therapies such as chemo- and radiotherapy.

Several pre-clinical and clinical studies have shown that combination of oncolytic viruses and radiation therapy may achieve additional or synergistic anti-tumor effects in *in vitro *and *in vivo *studies [109]. The experimental data demonstrated that ionizing radiation may also enhance viral replication and oncolysis within irradiated tumors.

Recently, the combination of oncolytic virotherapy with chemotherapy has shown that use of these two therapies with very distinct anti-tumor mechanisms may also lead to synergistic interactions [110]. Therefore, combination with radiation or chemotherapy may be a key to the optimization of the oncolytic viral therapy in dogs.

## 5. Ideal Oncolytic Virus for Canine Cancer Therapy

Improvement of replicating viral systems for canine cancer treatment is continuously being achieved, and many viruses are potentially useful for targeting canine cancer cells. However, an important question that still raises controversy is "which virus is best"? Several characteristics of oncolytic viruses for human cancer treatment have been described earlier [111,112]. There are no major variations as far as characteristics of ideal oncolytic viruses for canine cancer. An ideal oncolytic virus should demonstrate the following characteristics:

1. Efficient, safe and complete destruction of tumor tissue.

2. Selective replication in canine cancer cells.

3. Resistance against pre-existing immunity in canine populations.

4. Eliciting strong immune responses against tumor cells.

5. Propagation-deficient (safe) in immunocompromised patients.

6. Efficient clearance from the body preventing latent or recurrent infection.

7. No integration of viral genome into the canine genome.

8. Easily engineered to express antitumor agents.

9. Large recombinant gene carrying capacity.

10. Cost effectiveness and economy for widespread use in canine cancer patients.

11. Easy to monitor with respect to successful tumor colonization.

Many promising oncolytic viruses are being developed with convincing results in preclinical trials. However, it is unlikely that a virus will possess all the criteria of an 'ideal' oncolytic virus. In our opinion, vaccinia virus and adenovirus strains exhibit several characteristics of an ideal OV and show the most promising results in preclinical studies. In summary, similar to human cancer patients, OVs seem to hold promise in treatment of canine cancer.

## 6. Translation of Oncolytic Virotherapy from Dogs to Humans and the Reverse

Canine cancers share many features in common with human cancers including histological appearance, tumor genetics, molecular targets and response to conventional therapy [113-116]. In both species, tumor initiation and progression is influenced by similar factors like age, nutrition, sex and environmental exposure [117,118]. Dogs show as diverse cancers as seen in humans. These similarities are further seen in the development of therapeutics. Furthermore, carcinogenesis and tumor biologic behaviour in dogs have more features in common with humans than with laboratory rodents [119]. Despite evidence of oncolytic virus efficacy in mouse models of cancers, many viruses fail in human trials due to unacceptable toxicity or lack of efficacy [120]. Some strains of oncolytic viruses such as human adenovirus and vaccinia virus in general do not productively replicate in mouse cells. Thus, certain permissive cancer cells are grown in immunocompetent animals to study virus replication [121,122]. However, artificial establishment of these tumors as subcutaneous xenografts raises concern as to how well this model mimics their natural human counterparts. Hence, pet dogs with tumors are necessary models to demonstrate efficacy of OVs for human cancers. An alternative approach may be the use of species specific viruses in their natural host system. For example, application of Canine Adenovirus 2 in osteosarcoma of dog has shown to address the issue of tumor setting, efficient virus replication and oncolysis [30]. In this case, canine osteosarcoma resembles human osteaosarcoma at several levels including histopathology and metastatic behaviour [123]. However, Canine Adenovirus 2 shares similarities with human adenoviruses which are used as oncolytic agents for human osteosarcoma. The data from these studies are more reliable and may be helpful in designing human clinical trials. However, as far as veterinary medicine is concerned, development of oncolytic virotherapy for cancer to heal canine patients is of prime importance. Many of the treatment options used in veterinary medicine resemble protocols used to treat human cancer patients. In addition, public release of nearly 99% canine genome sequences provided a window of opportunity to expand the scope of comparative oncology. Comparison of canine genome sequences with the human genome suggests that around 19000 genes identified in the dog match to similar or orthologous genes in the human genome [124]. Taking into consideration the value of comparative oncology, data obtained from human clinical trials can be effectively transferred to canines.

## 7. Conclusions

Canine tumors are complex entities that continue to challenge modern veterinary medicine to develop more reliable cancer therapies. The field of oncolytic virotherapy is expanding and viruses continue to hold promise as an effective therapeutic approach for human cancers. Like in humans, oncolytic viruses replicate at the target site and spread within tumors to lyse neoplastic cells leading to decreased tumor burden in dogs. However studies of oncolytic viruses for canine cancer treatment are limited. It is not clear from past studies or from current understandings of potential oncolytic viruses which virus will be 'best' for canine cancer treatment. Nonetheless, preclinical studies with vaccinia and adenovirus for canine cancer therapy provided significant results which need exploration in canine clinical trials. Similarly, the therapeutic transgene expression capacity of these viruses will likely also need to be developed. Armed therapeutic viruses or genetically engineered viruses represent a very appealing tumor targeting approach and a novel opportunity to generate agents that could potentially cure canine cancers. Furthermore, studying dogs with cancer is likely to provide valuable information that is distinct from that generated in studies of rodent cancers alone. The importance of this opportunity must therefore be considered for the development of effective oncolytic therapy for canine as well as human cancers patients. It is hoped that collective efforts will contribute to the development of effective and safe viruses for both human and canine cancer therapy.

## List of abbreviations used

CDase: cytosine deaminase; CDV: Canine distemper virus; ECM: extracellular matrix; EGFR: Epidermal Growth Factor Receptor; GM-CSF: Granulocyte Macrophage Colony Stimulating Factor; GusA: beta-glucuronidase; HSP70: Heat Shock Protein 70; HSV: herpes simplex virus; IFNs: interferons; LacZ: beta-galactosidase; Ruc-GFP: *Renilla *luciferase-green fluorescent protein; LIVP: Lister strain of vaccinia virus; MV: Measles virus; NDV: Newcastle Disease Virus; OV: oncolytic virus; pRb pathway: retinoblastoma protein pathway; PTEN: phosphatase and tensin homologue deleted on chromosome 10; STAT3: Signal Transducer and Activator of Transcription 3 (gene); TAA: tumor associated antigens; *tk*: thymidine kinase gene; Ras: extracellular signal-regulated kinase; USDA: United States Department of Agriculture; VACV: vaccinia virus; VEGF: Vascular endothelial growth factor; VGF: Vaccinia Growth Factor.

## Competing interests

I. Gentschev and A.A. Szalay have interests in developing vaccinia virus as an oncolytic virus with Genelux Corporation, San Diego, USA. This work also was supported in part by grants from the Research and Development Division of Genelux Corporation, San Diego, USA. The funders had no role in study design, data collection and analysis, decision to publish, or preparation of the manuscript.

## Authors' contributions

SSP and IG critically reviewed the literature for the review. SSP, IG, IN, GO and AAS participated in conceiving the review and writing the manuscript. All the authors read and approved the final manuscript.
